# The "Complaints Book" at the South Infirmary, Cork

**Published:** 1911-09-09

**Authors:** Samuel N. Newson

**Affiliations:** Hon. Treasurer, South Infirmary.


*To the Editor of* The Hospital.

### THE "COMPLAINTS BOOK" AT THE SOUTH INFIRMARY, CORK.

*To the Editor of* The Hospital.

Dear— Sir,—The book to which you refer in Week's Work, August 26, page 548, is not what should be described as a complaint book, but a report book to see that all arrangements in the operating room are satisfactory. The idea emanated from a suggestion of one of the members of the surgical staff, and was adopted by the following resolution of the Joint Committee :—"It was decided to have a surgeon's report book kept in the operation theatre, and the house surgeon to write to the members of the surgical staff that the Joint Committee requested that each surgeon should, after operating, sign the book, stating whether the preparations of patients and instruments had been satisfactory and his instructions properly carried out by the resident doctors, students, and nurses. The book to be laid before the House Committee at their weekly meetings."—Yours faithfully,

Samuel N. Newson,

Hon. Treasurer, South Infirmary.

